# Host-directed microRNA-based intervention against intracellular *Staphylococcus aureus*: high-throughput screening identifies miR-4430, miR-147a, and miR-1249-5p as multifunctional antimicrobial candidates

**DOI:** 10.3389/fcimb.2026.1772100

**Published:** 2026-03-25

**Authors:** Pablo Castañera, Jesús Llano-Verdeja, Helena Á. Ferrero, Sergio Fernández-Martínez, Blanca Lorente-Torres, Fernando J. Pereira, Jesús F. Aparicio, Luis M. Mateos, Álvaro Mourenza, Michal Letek

**Affiliations:** 1 Departamento de Biología Molecular, Área de Microbiología, Universidad de León, León, Spain; 2 Departamento de Química y Física Aplicadas, Área de Química Analítica, Universidad de León, León, Spain; 3 Instituto de Biología Molecular, Genómica y Proteómica (INBIOMIC), Universidad de León, León, Spain; 4 Instituto de Desarrollo Ganadero y Sanidad Animal (INDEGSAL), Universidad de León, León, Spain

**Keywords:** antimicrobial, host-directed, intracellular, miRNA, RNA-seq, *Staphylococcus aureus*

## Abstract

**Background:**

The intracellular persistence of *Staphylococcus aureus* remains a major therapeutic challenge, enabling immune evasion and reducing the efficacy of antibiotics with limited intracellular activity. Host-directed therapies, particularly those based on microRNAs (miRNAs), represent a promising strategy to overcome these limitations.

**Methods:**

We performed a high-throughput screen of 2,469 human miRNA mimics in A549 epithelial cells infected with *S. aureus* USA300. Candidate miRNAs were prioritised using network centrality analysis and validated across different *S. aureus* strains and epithelial cell lines. RNA-seq profiling was conducted to examine host responses.

**Results:**

From this screen, ten candidates were identified, of which miR-4430, miR-1249-5p, and miR-147a consistently reduced intracellular bacterial burden and protected host cells. Transcriptomic analysis revealed complementary pathway-level programs: miR-4430 enhanced innate immune pathways, including STAT1- and PTAFR-associated signalling programs, while miR-1249-5p and miR-147a modulated extracellular matrix organisation and integrin-mediated adhesion, thereby interfering with bacterial entry.

**Discussion:**

These findings highlight a coordinated host defence strategy in which miR-4430 calibrates immune responses, while miR-1249-5p and miR-147a remodel host adhesion machinery. Together, they elicit complementary pressures that transiently reduce *S. aureus* intracellular proliferation and promote bacterial clearance. Our results underscore the therapeutic potential of miRNA-based host-directed interventions, which may be combined with conventional antibiotics to limit infection and resistance development.

## Introduction

*Staphylococcus aureus* is a facultative intracellular pathogen capable of causing a wide range of infections, from uncomplicated skin and soft tissue infections to life-threatening conditions such as endocarditis, surgical site infections, and pneumonia (Tong et al., 2015; Cheung et al., 2021). Its ability to survive and replicate within host cells allows it to evade both immune system responses and the action of antimicrobial agents that cannot penetrate host cell membranes. Combined with the rising prevalence of multidrug resistance, this intracellular lifestyle has rendered *S. aureus* a significant therapeutic challenge in recent years (Jansen et al., 2013; Stogios and Savchenko, 2020; Howden et al., 2023). Consequently, there is an urgent need to develop alternative therapeutic strategies to effectively combat *S. aureus* infections.

In this context, host-directed therapies (HDTs) have emerged as promising alternatives. By targeting host pathways rather than the pathogen itself, HDTs can circumvent intracellular bacterial protection mechanisms and reduce the selective pressure that drives the development of antimicrobial resistance (Kilinç et al., 2021; Cai et al., 2025). Among the most promising candidates for HDT strategies are microRNAs (miRNAs), which have recently demonstrated antimicrobial potential against intracellular pathogens, such as *Salmonella enterica* serovar Typhimurium and *Shigella flexneri* (Aguilar et al., 2020; Mourenza et al., 2022).

miRNAs are small, non-coding RNAs that play a pivotal role in post-transcriptional gene regulation. They modulate gene expression primarily through mRNA degradation or translational repression, affecting a wide array of biological processes across many organisms, including humans (Jonas and Izaurralde, 2015; Bartel, 2018). Approximately 2,500 human miRNAs have been described to date, each capable of regulating the expression of hundreds of target genes. It is estimated that up to 60% of the human transcriptome is influenced by miRNA activity (Friedman et al., 2009), underscoring their potential as regulators of host-pathogen interactions.

In this study, we performed high-throughput screening (HTS) of a 2,469-miRNA mimic library to identify candidates that modulate host gene expression and suppress *S. aureus* invasion and intracellular proliferation in human cells. Our goal was to evaluate the antimicrobial potential of miRNAs as HDT agents and elucidate their host transcriptional programs and downstream regulatory effects through transcriptome analysis using RNA sequencing (RNA-Seq). Among the identified miRNAs, miR-4430, miR-1249-5p, and miR-147a stood out for their ability to consistently preserve host cell viability and reduce bacterial burden across multiple *S. aureus* strains and host cell lines. Transcriptomic analyses revealed that miR-4430 likely exerts its effects by altering the expression of genes involved in immune responses, whereas miR-147a and miR-1249-5p appear to modulate genes associated with extracellular matrix organisation and cell adhesion, potentially interfering with bacterial internalisation. These findings highlight the potential of specific miRNAs as multifunctional host regulators and support their further exploration as host-directed strategies for the control of intracellular *S. aureus* infections.

## Methods

### Bacterial strains and growth conditions

*S. aureus* USA300 was used as the reference Community-Acquired Methicillin-Resistant *S. aureus* strain (CA-MRSA), while *S. aureus* NCTC 13626 was employed to evaluate the effect of selected miRNAs on Hospital-acquired Methicillin-resistant *S. aureus* (HA-MRSA) (Bravo-Santano et al., 2018). Both strains were routinely cultured in Brain Heart Infusion (BHI) broth at 37 °C with constant agitation at 200 rpm. Prior to each experiment, frozen bacterial aliquots were thawed and quantified to ensure accurate control of the Multiplicity of Infection (MOI).

### Cell Lines and growth conditions

The A549 cell line (American Type Culture Collection, Ref. CCL-185, USA) was used as a reference human epithelial cell line. Immortalised human Bronchial Epithelial Cells (BEC) expressing hTERT and BMI1 (Applied Biological Materials Inc., Cat. No. T0498, USA) were used to investigate the effects of miRNAs in different host cellular contexts. Both cell lines were maintained in Dulbecco’s Modified Eagle Medium (DMEM; Gibco, Spain) supplemented with 10% foetal bovine serum (FBS; Gibco, Spain) and 1× penicillin-streptomycin (Pen-Strep; Gibco, Spain) under standard conditions (37 °C, 5% CO_2_, and 95% humidity).

A549 and BEC cells were engineered to stably express the mCherry fluorescent protein by transduction with the p12-MMP-mCherry vector, as described previously (Bravo-Santano et al., 2018). To ensure that only successfully transduced cells persisted, hygromycin (Sigma-Aldrich) was added to the culture medium to selectively eliminate non-transduced cells. Cultures were maintained under standard conditions (37 °C, 5% CO_2_). The expression of mCherry was confirmed by measuring the fluorescence intensity using a VICTOR Nivo plate reader (PerkinElmer) with excitation and emission wavelengths of 580 and 625 nm, respectively (Lorente-Torres et al., 2025).

### High-throughput screening of miRNAs

A human library comprising 2,469 miRNA mimics, including mature isoforms, was obtained from Thermo Fisher Scientific (Ref. 4464074). Transfection was performed using Lipofectamine RNAiMAX (Thermo Fisher Scientific, USA) at a final concentration of 100 nM. Transfection efficiency was validated using a positive control siRNA targeting the essential *UBC* gene, which resulted in complete cell death. Synthetic *Caenorhabditis elegans* miRNA (cel-miR-231) was used as the negative control (Aguilar et al., 2020). A total of 10,000 A549 cells per well were seeded into black-walled, flat-bottom 96-well plates and incubated at 37 °C in a humidified atmosphere containing 5% CO_2_. Seventy-two hours post-transfection, the cells were infected with *S. aureus* USA300 (MOI = 10) and centrifuged at 800 × g. Cells were incubated for one hour under standard conditions (37 °C, 5% CO_2_, and 95% humidity), after which extracellular bacteria were eliminated by adding gentamicin (100 µg/mL) and vancomycin (5 µg/mL). Cell viability was assessed 24 h post-infection using a VICTOR Nivo multimode plate reader (PerkinElmer) to measure fluorescence from two independent biological replicates (n = 2). Constitutive mCherry fluorescence was used as a direct, metabolism-independent proxy for viable cell number. This readout was chosen to avoid confounding effects inherent to metabolic assays, which may be altered during intracellular infection independently of host cell death. In addition, this fluorescence-based approach enables non-invasive, longitudinal quantification of cell viability in intact monolayers, avoiding artefacts introduced by enzymatic detachment and fixation procedures required for flow-cytometry-based assays when working with intracellular pathogens (Lorente-Torres et al., 2025). Candidate miRNAs were prioritised using the miRNet 2.0 platform (Chang and Xia, 2023).

### Intracellular bacterial viability

The intracellular bacterial burden was quantified by counting colony-forming units (CFU). For this, 80,000 A549 cells were seeded per well in 96-well flat-bottom plates and incubated for 24 h under standard conditions. The cells were then infected with *S. aureus* USA300 (MOI = 10), centrifuged at 800× g, and incubated for 1 h. Extracellular bacteria were removed by treatment with gentamicin (100 µg/mL) and vancomycin (5 µg/mL). After 24 h of incubation, the cells were lysed with 0.1% Triton X-100 (Sigma-Aldrich, Spain), and serial dilutions were plated on BHI agar to determine CFUs. Plates were incubated at 37 °C before bacterial colonies were counted.

### RNA extraction, library preparation, and sequencing

Total RNA was isolated using the RNeasy Mini Kit (Qiagen) according to the manufacturer’s instructions. RNA quantity and purity were determined spectrophotometrically, and samples meeting quality requirements were processed for sequencing. To generate RNA-seq libraries compatible with host–pathogen transcriptome analysis, ribosomal RNA species were removed from total RNA preparations. The resulting RNA fraction was chemically fragmented and converted into double-stranded cDNA using standard reverse transcription procedures. cDNA molecules were then processed through end repair and adapter ligation, followed by size selection and amplification to produce sequencing libraries. Library construction was performed using a commercial RNA library preparation kit (Novogene NGS RNA Library Prep Set, PT042). Final libraries were quantified fluorometrically and by quantitative PCR, and fragment size distributions were assessed by capillary electrophoresis. Libraries passing quality control were pooled and sequenced on an Illumina NovaSeq X Plus system using paired-end reads. RNA-Seq analysis was performed on three independent biological replicates per condition (n = 3).

### RNA-seq analysis

Host raw RNA-Seq reads were aligned to the *Homo sapiens* reference genome using STAR aligner (Dobin et al., 2013). Gene-level count matrices were generated using the *featureCounts* tool. Downstream normalisation and differential gene expression analyses were performed using the *DESeq2* package in R (Love et al., 2014). A variance-stabilising transformation (VST) was applied before subsequent analyses (Zwiener et al., 2014). Functional enrichment analyses, including Gene Ontology (GO) and Reactome pathway analyses (Aleksander et al., 2023; Milacic et al., 2024), were performed using the *clusterProfiler*, *org.Hs.eg.db*, and *ReactomePA* R packages. Weighted Gene Co-expression Network Analysis (WGCNA) was conducted to identify gene modules associated with specific experimental conditions (Langfelder and Horvath, 2008). Raw and processed RNA-seq data have been deposited in the NCBI Gene Expression Omnibus (GEO) under accession GSE318734 and are available in the NCBI Sequence Read Archive (SRA) under accession SRP675283.

### Reverse transcription qPCR assay

Reverse transcription qPCR (RT-qPCR) was performed using the One-Step TB Green PrimeScript RT-qPCR Kit II (Takara Bio, Spain) on a QuantStudio 5 system (Applied Biosystems, USA). Primer sequences were obtained from the Harvard University PrimerBank database (Wang et al., 2012). Each reaction was performed in a final volume of 20 µL, following the manufacturer’s instructions. RNA samples were normalised to 50 ng/µL prior to addition to the reaction mixture. The thermocycling program consisted of an initial reverse transcription step at 42 °C for 5 min, followed by denaturation at 95 °C for 10 s, and then 40 cycles of amplification (95 °C for 5 s and 60 °C for 30 s). Relative gene expression levels were calculated using the 2^−ΔΔCt^ method, with GAPDH serving as the endogenous control. Fold-change values are presented in the figures, whereas statistical analyses were performed on the ΔCt values to validate the assumptions of parametric testing.

### Integration of shRNA screening and RNA-seq datasets

To integrate functional and transcriptomic evidence, filtered shRNA screening data obtained previously (Bravo-Santano et al., 2019a) were intersected with RNA-seq-derived differentially expressed genes (DEGs). The initial shRNA dataset was subjected to a stringent multi-step filtering process to identify robust and reproducible candidates. Briefly, shRNAs were required to display high consistency across biological replicates (consistency score ≥ 0.8), meet statistical significance criteria, and exhibit a biologically relevant effect size (fold change ≥ 0.5 or ≤ −1). The resulting high-confidence set of shRNA-supported genes (n = 5344) was intersected with DEGs identified in A549 cells transfected with miR-4430, miR-147a, or miR-1249-5p and infected with *S. aureus*. Intersections were computed independently for each condition to identify genes supported by both transcriptional modulation and functional evidence. Overlap relationships among datasets were visualized using an UpSet plot.

### Statistical analysis

Statistical analyses, excluding RNA-seq data, were performed using GraphPad Prism version 8 (GraphPad Software). The normality of the data distribution was assessed using the Shapiro–Wilk test. Parametric data were analysed using unpaired t-tests, and non-parametric data were evaluated using the Mann–Whitney U test. Depending on the experiment, results are reported as either mean ± standard deviation (SD) or mean ± standard error of the mean (SEM), based on technical duplicates or biological replicates. Statistical significance was set at *p* < 0.05.

### Ethical approval

The study was approved by the Ethics Committee of the *Universidad de León* (ETICA-ULE-060–2021 and ETICA-ULE-003-2023) for research involving biosafety level 2 pathogens and genetically modified human cell lines.

## Results

### HTS of miRNA mimics against *S. aureus* USA300 intracellular infection

**Figure 1:**
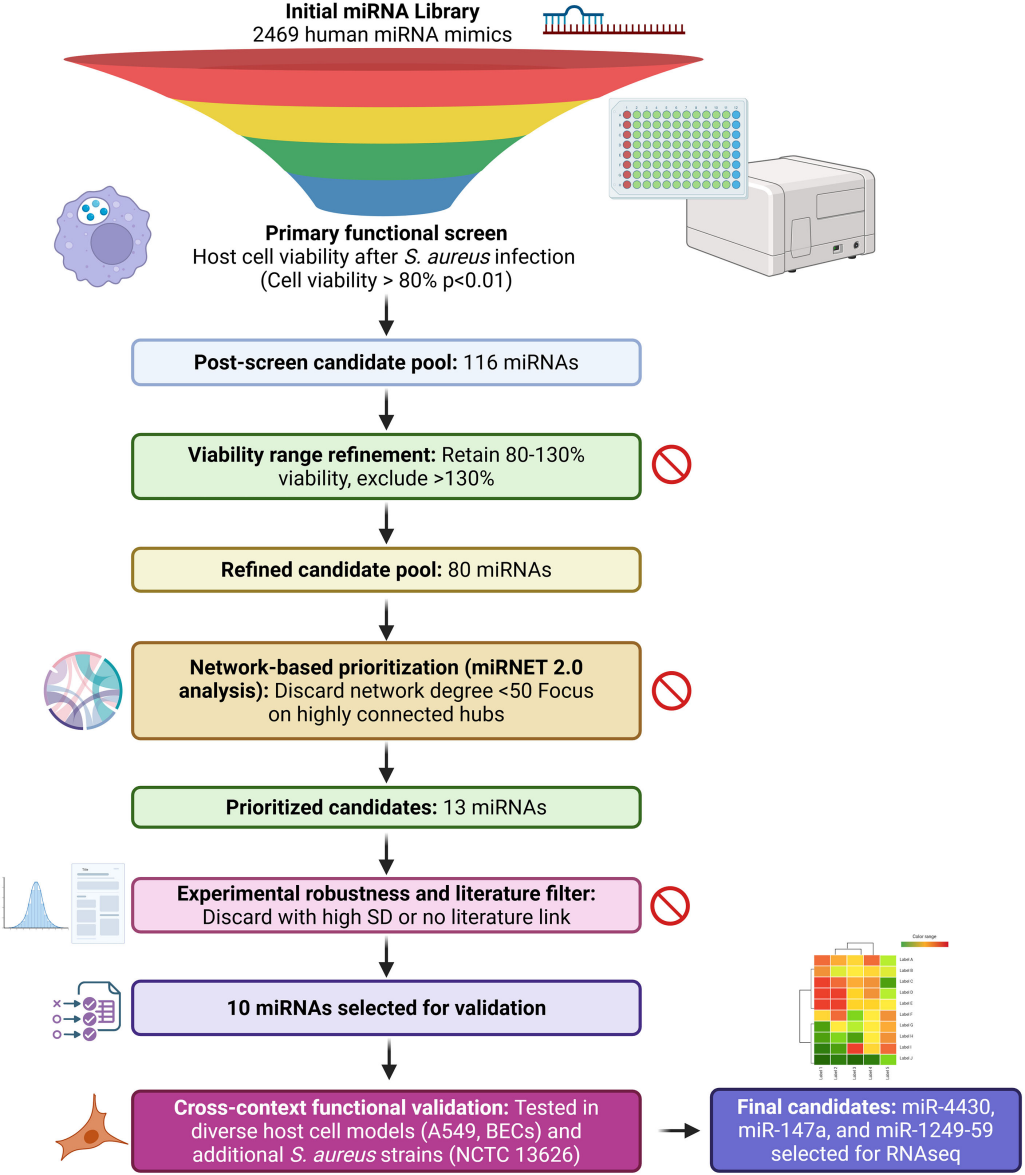
Stepwise prioritisation pipeline to identify host-directed miRNAs that preserve host cell viability during *S. aureus* infection. A high-throughput functional screen of 2,469 human miRNA mimics was performed to assess host cell viability following *S. aureus* USA300 infection. miRNAs preserving host cell viability (>80%, *p* < 0.01) were retained, yielding an initial post-screen candidate pool of 116 miRNAs. Candidates were further refined by excluding miRNAs inducing excessive viability (>130%), resulting in 80 retained miRNAs. Network-based prioritisation using miRNet 2.0 was then applied to focus on highly connected regulatory hubs, discarding miRNAs with low network degree (<50) and reducing the pool to 13 candidates. An additional experimental robustness and literature-based filter removed miRNAs with high variability or lacking prior biological support, yielding 10 miRNAs selected for validation. These candidates were functionally validated across distinct host cell models (A549 epithelial cells and primary bronchial epithelial cells, BECs) and an additional *S. aureus* strain (NCTC 13626). Three miRNAs (miR-4430, miR-147a, and miR-1249-5p) consistently preserved host cell viability across conditions and were selected for downstream transcriptomic (RNA-seq) analysis. Created with Biorender.

**Figure 2:**
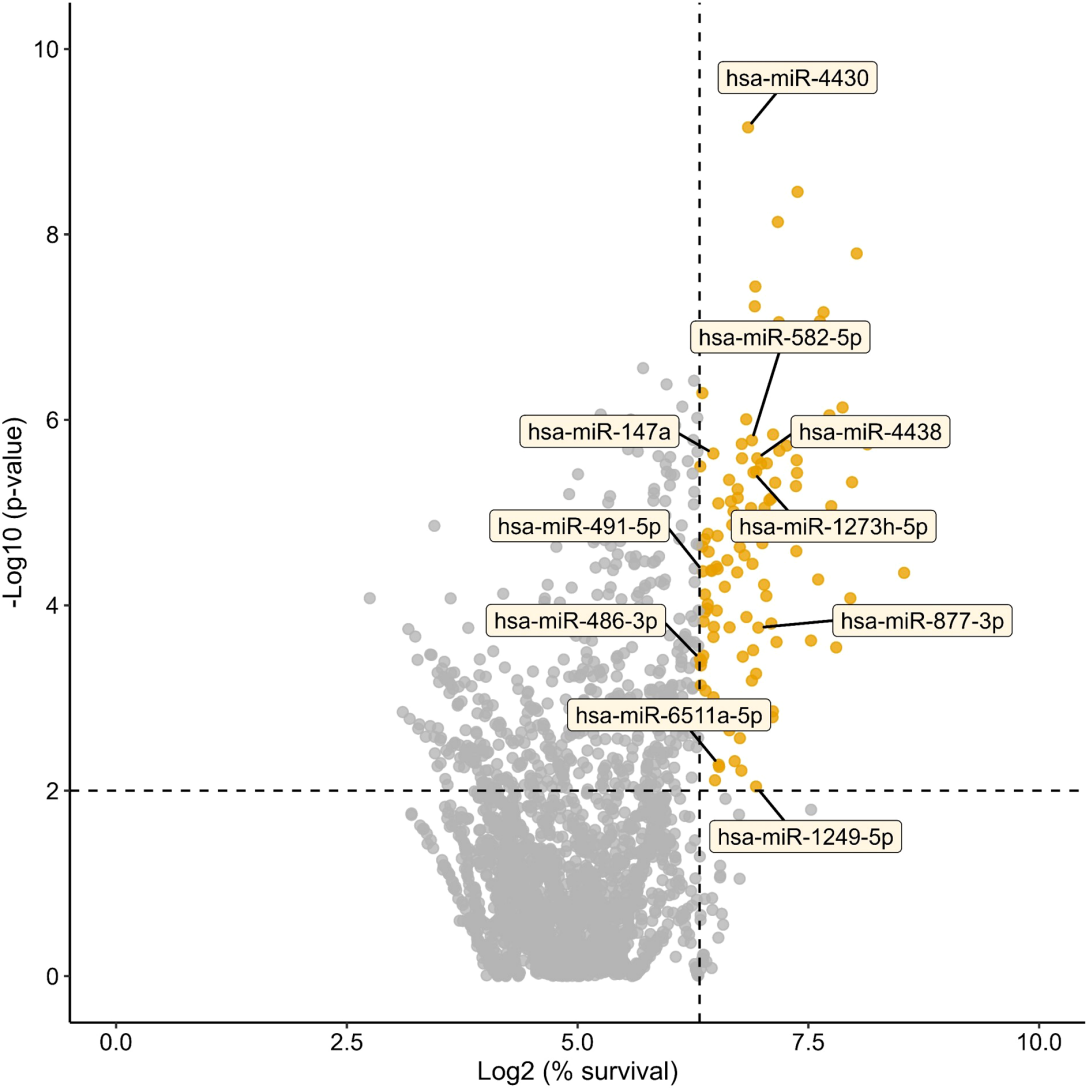
High-throughput screening of human microRNAs affecting host cell viability upon *S. aureus* infection. Results of a high-throughput screening of 2,469 human miRNA mimics in A549 epithelial cells infected with *S. aureus* USA300. A total of 116 miRNAs (4.7%) significantly modulated host cell viability (*p* < 0.01), displaying viability values above 80% relative to infected, non-transfected controls. Data represent two independent biological replicates (n = 2).

HTS was performed to identify miRNAs with potential regulatory effects on intracellular infection by *S. aureus* USA300 in A549 epithelial cells constitutively expressing the mCherry fluorescent protein (**Figure 1**). A total of 2,469 human miRNA mimics were transfected individually, and their effects on host cell viability post-infection were evaluated by quantifying fluorescence, which is a reliable method for assessing cell viability (Lorente-Torres et al., 2025). From the initial miRNA library, 116 miRNAs (p < 0.01) exhibited cell viability values above 80% relative to the infected, non-transfected control group. Among these, 80 miRNAs maintained cell viability within the 80–130% range and remained statistically significant (*p* < 0.01) and were therefore selected for downstream analysis (**Figures 1**, **2**, **Supplementary Table 1**).

These 116 miRNAs were subsequently analysed using the miRNet 2.0 platform to explore their potential connectivity and functional relevance. To prioritise those most likely involved in regulatory networks, miRNAs with a network degree lower than 50 were discarded, resulting in a subset of 13 candidates (**Supplementary Figure 1**; **Supplementary Table 2**). This step removed approximately 87.6% of the previously filtered candidates, allowing us to focus on highly connected and potentially biologically relevant miRNAs (Chang and Xia, 2023).

Further refinement was applied to these 13 candidates based on their experimental robustness and prior evidence in infectious contexts. Specifically, miRNAs that showed average viability values close to the lower acceptance threshold (80%) combined with high standard deviation values— sufficient to potentially place their true effect outside the viability range—were excluded. Additionally, if no literature-supported role could be identified for these miRNAs in infection or immune response, they were de-prioritized. In total, three miRNAs were excluded during this step based on these combined criteria, leading to a final list of ten candidates for downstream functional validation experiments (**Figure 1**).

The final list of selected miRNAs included miR-1273h-5p, miR-6511a-5p, miR-4430, miR-877-3p, miR-491-5p, miR-1249-5p, miR-4438, miR-582-5p, miR-486-3p, and miR-147a. Several of these candidates have previously been implicated in host-pathogen interactions or immune-related processes. In particular, miR-1273h-5p has been identified in latent *Mycobacterium tuberculosis* infection in macrophages (Meng et al., 2014), whereas miR-4430 has been associated with inflammation-related mechanisms (Wang et al., 2018). miR-877-3p shows differential expression between latent and active tuberculosis cases (Aulicino et al., 2015), and miR-1249-5p has been linked to *M. tuberculosis* infection in macrophages (Wu et al., 2016). miR-582-5p has been reported to undergo arm-switching under infection conditions (Siddle et al., 2015), and its expression is affected during *M. tuberculosis* infection (Von Both et al., 2018). Furthermore, miR-486-3p and miR-4438 have been associated with host responses to *Escherichia coli* and *M. tuberculosis* (Jin et al., 2014; Zhang et al., 2014; Taofeek et al., 2025). Both miR-6511a-5p and miR-491-5p have been linked to viral infections (Ishida et al., 2011; Seong et al., 2020). Finally, miR-147a has been shown to play a role in inflammation triggered by lipopolysaccharide (LPS), a key mediator of infection dynamics (Liu et al., 2009).

### Validation of selected miRNAs

To validate and further characterise the regulatory potential of the selected miRNAs in diverse infection contexts, functional assays were performed using an alternative *S. aureus* strain and a different human epithelial cell line. Specifically, the selected miRNA candidates were evaluated in A549 cells infected with *S. aureus* USA300, a community-acquired MRSA strain, as well as in A549 cells infected with *S. aureus* NCTC 13626, a hospital-acquired MRSA strain (Bravo-Santano et al., 2019b). In addition, their effects were assessed in immortalised human bronchial epithelial cells (BEC), which are non-tumorigenic in origin.

**Figure 3:**
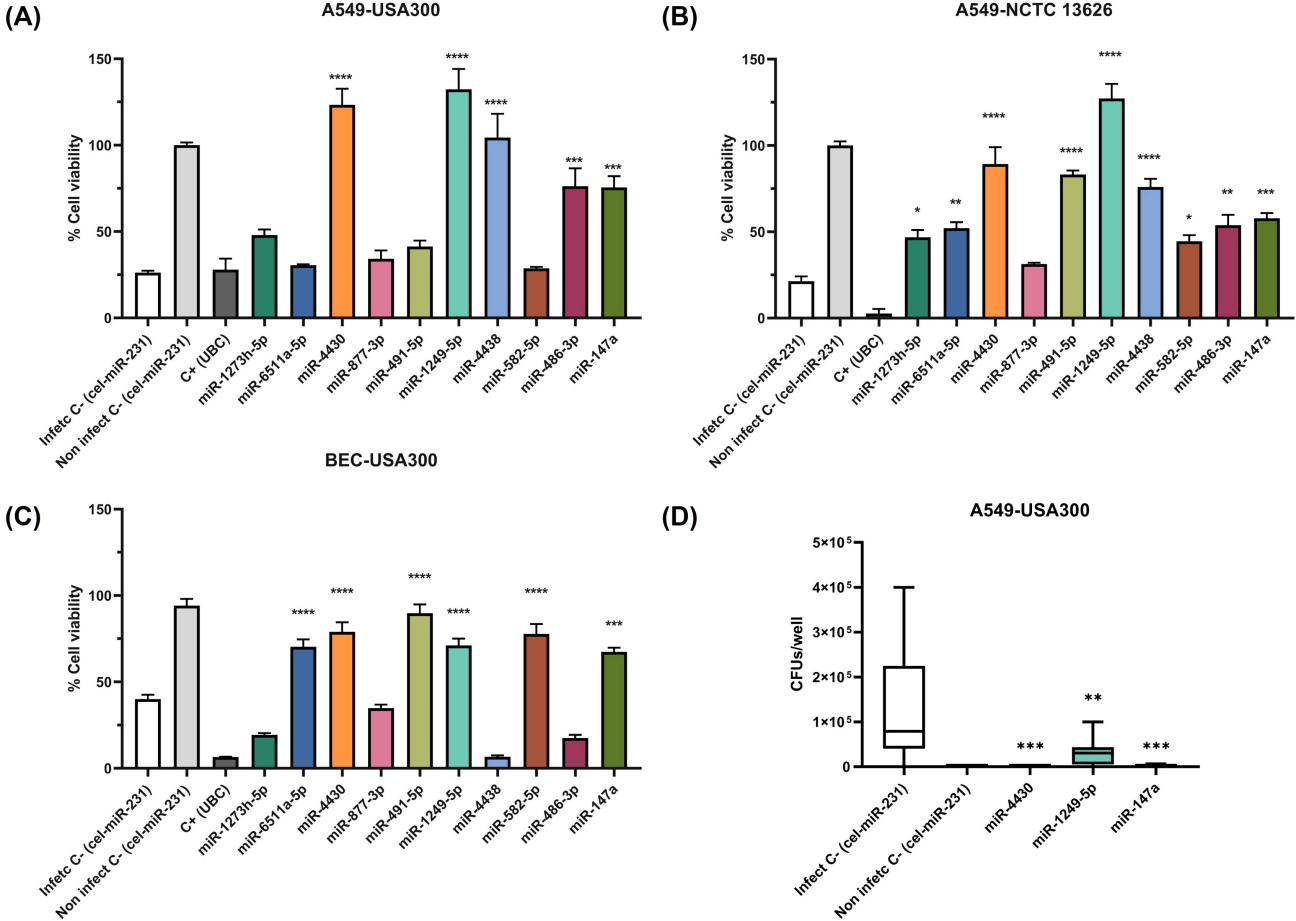
Validation of selected miRNAs in *S. aureus* strains and host cell lines. **(A)** Effect of the 10 prioritised miRNAs on host cell viability in A549 cells infected with *S. aureus* USA300. **(B)** Validation of candidate miRNAs in A549 cells infected with the hospital-acquired MRSA strain *S. aureus* NCTC 13626. **(C)** Effect of selected miRNAs in immortalised human bronchial epithelial cells (BEC) infected with *S. aureus* USA300. **(D)** Quantification of intracellular bacterial burden in A549 cells transfected with candidate miRNAs and infected with *S. aureus* USA300, expressed as colony-forming units (CFU). Data in panels **(A–C)** represent four independent biological replicates (n = 4), while data in panel D represent three independent biological replicates (n = 3). miR-4430, miR-1249-5p, and miR-147a consistently improved host cell viability and reduced bacterial burden across different *S. aureus* strains and host cell types, supporting their role as robust host-directed antimicrobial candidates. * p < 0.05; ** p < 0.01; *** p < 0.001; **** p < 0.0001.

Notably, five of the ten miRNAs—miR-4430, miR-1249-5p, miR-4438, miR-486-5p, and miR-147a—demonstrated robust protective effects, significantly preserving host cell viability in both *S. aureus* USA300 and NCTC 13626 infections in A549 cells (**Figures 3A, B**). Furthermore, three miRNAs—miR-4430, miR-1249-5p, and miR-147a—exhibited consistent protective activity in both A549 and BEC infected with the *S. aureus* USA300 strain (**Figures 3A, C**). These three candidates were the only ones from the initial panel to show efficacy across all tested conditions, reducing infection-associated cytotoxicity and enhancing host cell viability, regardless of the strain or cell line (**Figures 3A–C**). Additionally, these three miRNAs demonstrated the ability to promote bacterial clearance in A549 cells infected with *S. aureus* USA300, as evidenced by CFU quantification (**Figure 3D**).

To address concerns regarding dose dependency, kinetics, and potential off-target effects associated with high miRNA mimic concentrations, we performed dose-response and time-course analyses for the three selected miRNAs. As shown in **Supplementary Figure 2**, miRNA-mediated preservation of host cell viability followed a graded, dose-dependent pattern at earlier time points post-infection. However, this protective effect was not sustained over time, and host cell viability decreased at later time points, indicating that the observed phenotype is transient rather than permanent. This behaviour is inconsistent with nonspecific activation of RNA-sensing pathways, which typically display rapid, switch-like responses (Olejniczak et al., 2009). Instead, the graded, time-limited nature of the phenotype supports a regulated, host-directed effect that modulates infection outcomes without inducing sustained innate immune overstimulation.

### Host transcriptomic analysis of *S. aureus*–infected cells treated with miRNAs

To gain a deeper understanding of the genes regulated by the three miRNAs that demonstrated robust host-protective effects across different infection contexts, we performed a transcriptome profiling of A549 epithelial cells infected with *S. aureus* USA300 and pre-transfected with the corresponding miRNAs (miR-4430, miR-1249-5p, and miR-147a; **Supplementary Tables 3**–**S5**). Among the conditions tested, A549 host cells transfected with miR-1249-5p exhibited the highest number of uniquely differentially expressed genes, indicating a broader transcriptional impact than that of the other miRNAs (**Supplementary Figure 3A**). In contrast, the transcriptome of A549 cells transfected with miR-4430 appeared more clustered, indicating a higher degree of co-regulation and consistency in the genes affected by this specific miRNA (**Supplementary Figure 3B**). Moreover, the Differentially Expressed Genes (DEG) profiles revealed that A549 cells transfected with miR-4430 presented more pronounced changes in specific genes upon *S. aureus* USA300 infection (**Supplementary Figure 4A**) than the DEG patterns observed in the miR-1249-5p and miR-147a conditions (**Supplementary Figure 4B, C**, respectively).

### miR-4430 modulates immunity-related gene programs during *S. aureus* infection

The promising effects of miR-4430 on maintaining host cell viability and promoting intracellular bacterial clearance prompted a deeper investigation into the most enriched and downregulated functional pathways associated with its expression in this study. Given that different functional analysis tools may yield distinct outputs, we employed a combined approach using multiple resources to ensure robust and comprehensive transcriptomic assessment. Specifically, we conducted Gene Ontology (GO) and Reactome pathway enrichment analyses.

GO analysis revealed that miR-4430 significantly modulated immune-related pathways. In the Biological Process (BP) category, terms such as *defence response to virus*, *response to type I interferon*, and *positive regulation of cytokine production* were prominently enriched (**Supplementary Figure 5A**). In the Cellular Component (CC) category, *blood microparticles* were a significant term (**Supplementary Figure 5B**), a component known to include platelet-derived microparticles that play important roles in immune modulation and host defence (Italiano et al., 2010).

**Figure 4:**
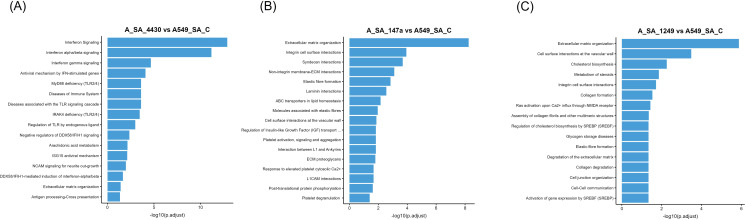
Reactome pathway enrichment analysis of host transcriptomic changes induced by candidate miRNAs during *S. aureus* infection. **(A)** miR-4430 treatment enriched immune-related pathways, including interferon signalling and antiviral mechanisms, by IFN-stimulated genes. **(B)** miR-147a treatment enriched pathways associated with extracellular matrix organisation and integrin-mediated adhesion. **(C)** miR-1249-5p treatment enriched pathways linked to cell adhesion and cholesterol biosynthesis. These analyses indicate that miR-4430 reinforces innate immune defences, whereas miR-147a and miR-1249-5p remodel adhesion and membrane pathways, highlighting the complementary mechanisms of host protection against *S. aureus*.

Reactome pathway analysis further supported these findings, revealing that immune regulatory pathways, such as *interferon signalling*, *antiviral mechanisms by IFN-stimulated genes*, and *MyD88 deficiency*, were significantly affected in miR-4430–treated A549 cells infected with *S. aureus* USA300 (**Figure 4A**). Additionally, Weighted Gene Co-expression Network Analysis identified a significant gene co-expression network strongly correlated with miR-4430 treatment, further supporting its regulatory impact on immunity-related genes during *S. aureus* infection (**Supplementary Figure 6**).

**Figure 5:**
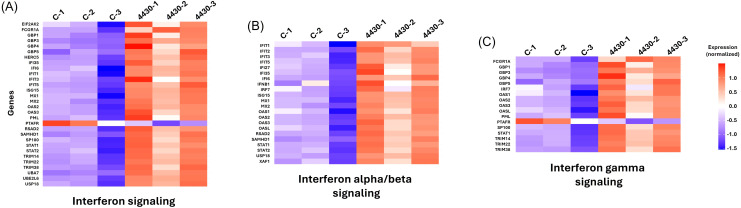
Reactome pathway enrichment of immune signalling pathways modulated by miR-4430 during *S. aureus* infection. **(A)** Interferon signalling pathway, showing altered expression of genes such as *STAT1* and *PTAFR*. **(B)** Interferon alpha/beta signalling pathway with significant modulation of antiviral response genes. **(C)** Interferon-gamma signalling pathway, highlighting *STAT1* as a central regulator. These data demonstrate that miR-4430 promotes the activation of interferon-mediated signalling networks, reinforcing innate immune responses to restrict intracellular *S. aureus*.

These results suggest that the protective effect of miR-4430, which preserves host viability and facilitates bacterial clearance, is mechanistically linked to its modulation of immune response genes. Among the differentially expressed genes, several have been identified as critical mediators of *S. aureus* pathogenesis. Importantly, these genes should be interpreted as downstream transcriptional responses to miRNA treatment rather than direct miRNA-mRNA targets. In particular, *PTAFR* was found within the Reactome categories of *interferon signalling* and *interferon gamma signalling* (**Figures 5A, C**) and has been implicated in the regulation of immune responses during *S. aureus* infection (Iovino et al., 2013). Similarly, *STAT1*, a central transcription factor in innate immunity, was present in multiple enriched pathways, including *interferon signalling*, *interferon alpha/beta signalling*, and *interferon gamma signalling* (**Figure 5**), and has been shown to play a key role in controlling infection dynamics (Parker and Prince, 2012; Bravo-Santano et al., 2019a).

### *S. aureus* internalisation is modulated by miR-147a and miR-1249-5p

GO analysis indicated that miR-147a modulates key pathways involved in cellular adhesion, which are crucial for *S. aureus* internalisation. The enriched biological processes included *cell–substrate adhesion* and *extracellular matrix organization* (**Supplementary Figure 7A**). In the cellular component category, terms such as *collagen-containing extracellular matrix* and *protein complex involved in cell adhesion* were significantly represented (**Supplementary Figure 7B**). Similarly, the molecular function category showed enrichment for *integrin* and *proteoglycan binding* (**Supplementary Figure 7C**).

In parallel, GO analysis of *S. aureus* USA300-infected A549 cells treated with miR-1249-5p also revealed significant modulation of *cell–substrate adhesion* pathways and processes linked to *cholesterol biosynthesis/metabolism* (**Supplementary Figure 8**). This is particularly noteworthy because lipid raft membrane domains, dynamic cholesterol- and sphingolipid-rich microdomains implicated in bacterial uptake, are influenced by cholesterol metabolism (Mergani et al., 2021; Goldmann et al., 2024).

**Figure 6:**
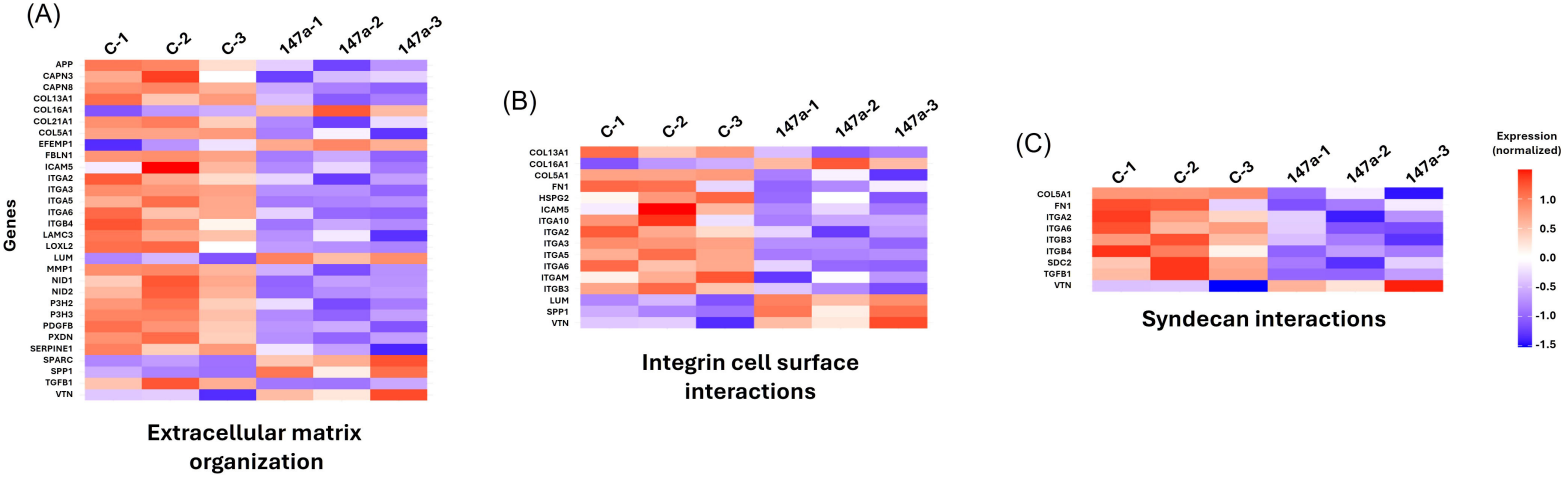
Reactome pathway enrichment of adhesion-related processes modulated by miR-147a during *S. aureus* infection. **(A)** Extracellular matrix organisation pathway showing the downregulation of *FN1* (fibronectin). **(B)** Integrin cell surface interaction pathway, highlighting the downregulation of *ITGA5* (integrin α5). **(C)** Syndecan interaction pathway, which also involves *FN1*. These results indicate that miR-147a interferes with fibronectin–integrin-mediated bacterial internalisation, thereby limiting *S. aureus* entry into host cells.

**Figure 7:**
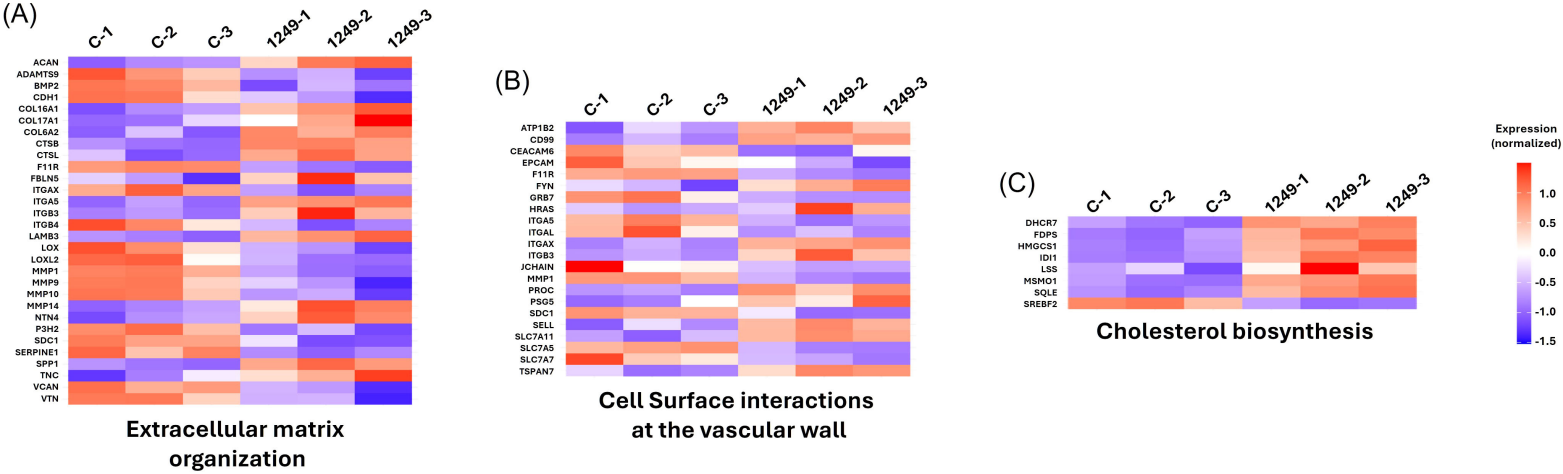
Reactome pathway enrichment of adhesion-related processes modulated by miR-1249-5p during *S. aureus* infection. **(A)** Extracellular matrix organisation pathway showing the altered regulation of adhesion-associated genes. **(B)** Cell surface interactions in the vascular wall pathway, highlighting the downregulation of *ITGA5* (integrin α5). **(C)** Cholesterol biosynthesis and metabolism–related pathways, consistent with alterations in membrane composition linked to bacterial entry processes. Together, these analyses indicate that miR-1249-5p modulates extracellular matrix, integrin, and lipid-associated host pathways, consistent with reduced *S. aureus* internalisation through downstream remodelling of epithelial adhesion mechanisms.

Consistently, Reactome pathway analysis supported these findings, showing that *cell adhesion-related pathways* were among the top enriched categories in both miRNA treatments (miR-147a: **Figures 4B**, **6**; miR-1249-5p: **Figures 4C**, **7**). Additionally, *cholesterol metabolism* pathways were significantly enriched following miR-1249-5p treatment (**Figure 7C**).

Importantly, analysis of differentially expressed genes within the enriched Reactome pathways revealed the downregulation of *ITGA5* in both miR-147a- and miR-1249-5p-treated cells, suggesting indirect regulation of host entry pathways. Specifically, *extracellular matrix organisation* and *integrin cell surface interactions* were enriched following miR-147a treatment (**Figures 6A, B**), whereas *extracellular matrix interactions* and *cell surface interactions at the vascular wall* were enriched following miR-1249-5p treatment (**Figures 7A, B**). *ITGA5*, which encodes integrin alpha-5 protein, is an essential mediator of *S. aureus* internalisation. Its loss impairs bacterial entry by removing the integrin-binding site required for host cell invasion (Morgenstern et al., 2024). Moreover, *FN1* was identified as a differentially expressed gene in both *integrin cell surface interactions* and *syndecan interactions* in miR-147a-treated infected cells (**Figures 6B, C**). *FN1* encodes fibronectin, a known bridging molecule exploited by *S. aureus* for internalisation, and its knockdown is associated with reduced bacterial uptake by keratinocytes (Miyake et al., 2022).

### RNA-seq validation

**Figure 8:**
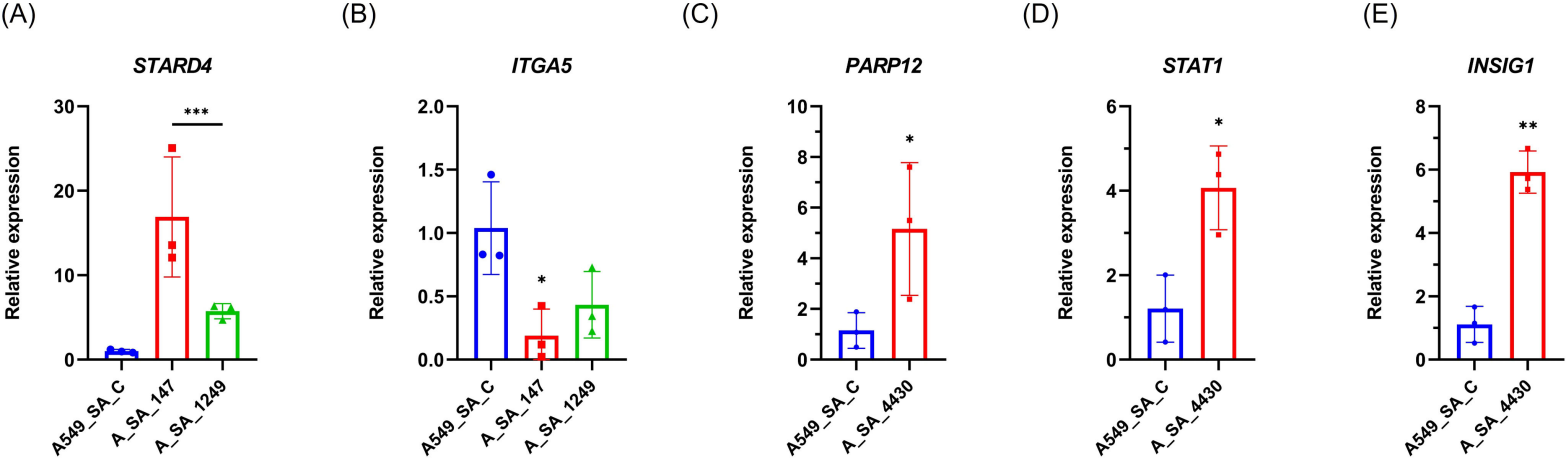
Validation of RNA-seq results by RT-qPCR in A549 cells transfected with miR-4430, miR-147a, or miR-1249-5p and infected with *S. aureus* USA300. **(A)** The Expression of *STARD4* was significantly upregulated in cells treated with miR-147a and miR-1249-5p. **(B)** Expression of *ITGA5* was significantly downregulated following treatment with miR-147a and miR-1249-5p. **(C–E)** Expression of *PARP12*, *STAT1*, and *INSIG1* was significantly upregulated in cells treated with miR-4430. These results confirm the RNA-seq findings, supporting the downstream transcriptional effects of miR-4430 on immune signalling, whereas miR-147a and miR-1249-5p are associated with downstream modulation of adhesion- and lipid-related genes that influence *S. aureus* internalisation. Data represent three independent biological replicates (n = 3). * p < 0.05; ** p < 0.01; *** p < 0.001.

RNA-seq results were validated using RT-qPCR analysis. Consistently, several differentially expressed genes identified by RNA-seq were confirmed. Specifically, a significant upregulation of *STARD4* was observed in infected A549 cells treated with miR-147a or miR-1249-5p compared to the control, in agreement with the RNA-seq data (**Figure 8**, **Supplementary Figure S9**). Similarly, *ITGA5* expression was significantly downregulated following treatment with miR-147a and reduced upon treatment with miR-1249-5p, corroborating the RNA-seq findings. Furthermore, treatment with miR-4430 resulted in a significant upregulation of *PARP12, STAT1*, and *INSIG1* in infected A549 cells compared to the controls, consistent with the transcriptomic analysis (**Figures 8**, **Supplementary Figure S9**).

To assess whether inhibition of individual candidate miRNAs produced deleterious or nonspecific effects during infection, we performed complementary experiments using miRNA-specific antagomirs. As shown in **Supplementary Figure 10**, antagomir-mediated inhibition of the selected miRNAs did not significantly alter host cell viability in A549 cells infected with *S. aureus* USA300 when compared with cells transfected with a non-targeting control miRNA. These results indicate that inhibition of individual miRNAs does not exacerbate infection-associated cytotoxicity under the conditions tested.

To further integrate transcriptomic changes with prior functional evidence, RNA-seq–derived differentially expressed genes were intersected with a filtered shRNA screening dataset previously generated in infected host cells. This integrative analysis revealed a subset of genes supported by both transcriptional modulation and functional loss-of-function evidence (**Supplementary Figure 11**). Among these, *ITGA5* emerged as a consistently regulated gene across miR-147a– and miR-1249-5p–treated conditions, supporting its relevance as a downstream host factor associated with altered bacterial internalization.

## Discussion

Previous high-content loss-of-function screens in infected host cells consistently pointed to entry and intracellular *S. aureus* survival hinging on host membrane signalling and Ca²^+^ homeostasis. An shRNA screen focused on phosphoinositide 5-phosphatases identified synaptojanin-1 (SYNJ1) as a restriction factor: depleting SYNJ1 elevates local PI(4,5)P_2_, promotes talin recruitment and enlarged invaginations, and increases integrin-mediated uptake of *S. aureus*—implicating PI(4,5)P_2_ turnover as a gatekeeper for invasion (Shi et al., 2024). A genome-wide shRNA screen independently converged on Ca²^+^ signalling as central to pathogenesis: intracellular *S. aureus* drive endoplasmic reticulum Ca²^+^ depletion and cytosolic/mitochondrial Ca²^+^ overload, activating calpains/caspases and host-cell death that enables exit and spread (Stelzner et al., 2020). Complementing these mechanistic hits, a pooled 16,000-gene shRNA screen flagged host pathways co-opted during infection (autophagy, actin/MAPK/TLR signalling) and singled out TRAM2–SERCA as actionable; Thapsigargin (SERCA inhibitor) curtailed intracellular MRSA, illustrating host-directed therapy (HDT) potential (Bravo-Santano et al., 2019a). In addition, miRNA surveys show that host miRNAs broadly reprogram inflammatory, innate and adaptive responses during *S. aureus* infection, providing an additional layer that can either favour intracellular survival or enhance clearance, and offering biomarker and HDT entry points (Mirzaei et al., 2020).

Through high-throughput screening and multi-context validation, we identified here three miRNAs—miR-4430, miR-1249-5p, and miR-147a—that consistently preserved host cell viability and reduced bacterial burden across different *S. aureus* strains and host cell lines. This robust antimicrobial effect across diverse infection contexts is particularly notable, as miRNAs are often context-dependent and may exert contrasting roles depending on the host cell type or infection model (Rothchild et al., 2016). Furthermore, multiple studies report strong cell-type and strain effects in *S. aureus* infection—host responses recorded in one epithelial or phagocytic model often do not replicate in another, and highly invasive/persistent strains (e.g., USA300) behave differently from less invasive lineages (e.g., Newman)—underscoring that HDT hits may fail to generalize across models and should be validated across cell contexts and strain backgrounds (Chang et al., 2025).

In our data, miR-4430 primarily appears to tune the inflammatory set-point of the infected cell—dampening damaging host responses while preserving antibacterial capacity—whereas miR-147a and miR-1249-5p chiefly modulate bacterial internalisation, acting on host entry machinery (integrin/focal-adhesion signalling). This division-of-labour is remarkable for HDT because it yields orthogonal levers that can, in principle, be combined: miR-4430 to calibrate an immune response, and miR-147a/miR-1249-5p to curb uptake and reduce host cell invasion. This aligns with our cross-context tests showing that, despite known cell-type and strain dependencies in *S. aureus* infection, each miRNA exerts a pathway-specific phenotype. Pairing an immune-tone miRNA (miR-4430) with an entry-blocking miRNA (miR-147a or miR-1249-5p) could be a rational HDT strategy.

Importantly, in this study, differentially expressed genes identified by RNA-seq are interpreted as downstream-regulated genes rather than direct miRNA targets. While miRNAs exert their primary effects through sequence-specific interactions with target mRNAs, transcriptome-wide changes also reflect secondary and tertiary regulatory cascades. Dissecting direct miRNA–mRNA interactions for individual candidates will require dedicated mechanistic studies, which fall outside the scope of this work and represent an important avenue for future research.

Nevertheless, the host pathways highlighted by our integrative analysis converge on mechanisms that are extensively documented in the literature as central to *S. aureus* internalisation and intracellular control. STAT1 has been repeatedly implicated in host responses to intracellular *S. aureus*. Previous work shows that STAT1-dependent signalling contributes to phagosome maturation and shapes interferon-driven transcriptional programs that influence intracellular bacterial control (Martin et al., 2009; Zhu et al., 2015; Wu et al., 2021; Rad et al., 2025). These studies support a role for STAT1-associated networks in determining infection outcome and are fully consistent with the modulation of STAT1-linked signatures observed in our host transcriptomic data following miR-4430 treatment.

Moreover, the fibronectin–integrin α5β1 axis has long been recognised as a key entry route exploited by *S. aureus*, whereby fibronectin acts as a bridging molecule between bacterial surface adhesins and host integrins to promote uptake into epithelial cells (Sinha et al., 1999; Kintarak et al., 2004; Borisova et al., 2013; Robertin et al., 2023; Morgenstern et al., 2024). These studies demonstrate that disruption of *FN1* or *ITGA5* expression markedly impairs bacterial internalisation across multiple host cell types, underscoring the functional relevance of this pathway during infection.

Importantly, this represents, to our knowledge, one of the first demonstrations that host microRNAs can restrict *S. aureus* infection through downstream remodelling of epithelial adhesion and internalisation pathways. While previous miRNA studies in *S. aureus* infection have largely focused on immune regulation, inflammatory signalling, or biomarker discovery (Mourenza et al., 2022), none have demonstrated a role for miRNAs in controlling integrin- and extracellular matrix–dependent bacterial entry. The identification of miR-147a and miR-1249-5p as candidates linked to downstream regulation of ITGA5- and FN1-associated pathways, therefore, reveals a previously unrecognised layer of host-directed control over *S. aureus* internalisation.

A key challenge for RNA-based therapeutics is delivery, as unmodified oligonucleotides exhibit limited stability, uptake, and endosomal escape (Maziec et al., 2025). Advances from antiviral RNA therapeutics—including optimized chemistries (e.g., LNA, PMO), lipid nanoparticle formulations, and conjugation strategies—have substantially improved intracellular delivery while reducing innate immune activation (Chow et al., 2020; Sung and Kim, 2020; Feng et al., 2021; Friedrich and Aigner, 2022; Paunovska et al., 2022; Ingle and Fang, 2023). Importantly, pulmonary delivery routes have demonstrated clinical feasibility for RNA cargos, suggesting particular promise for respiratory infections (Chow et al., 2020). Together, these advances indicate that miRNA delivery is tractable and support further exploration of miRNA-based host-directed strategies against intracellular *S. aureus*.

Collectively, our results define a coordinated miRNA-mediated host defence in which miR-4430 calibrates innate immune signalling. At the same time, miR-147a and miR-1249-5p remodel integrin/ECM-dependent entry pathways. Our results suggest that orthogonal host-directed interventions can be paired with standard antibiotics to limit host cell invasion and intracellular proliferation, with potential implications for resistance management.

## Data Availability

The original contributions presented in the study are publicly available. This data can be found here: https://www.ncbi.nlm.nih.gov/geo/query/acc.cgi?acc=GSE318734 with accession number GSE318734.

## Supplementary material

Supplementary Table S1 High-throughput screening of host miRNAs against intracellular *S. aureus*. Summary of miRNAs tested in the HTS, including plate number, miRNA name, percentage of host cell survival after infection, standard deviation, probability values from t-tests, nucleotide sequence, negative log10 of the p-value, and log2-transformed survival percentages.

Supplementary Table S2 List of miRNAs identified in the high-throughput screen as significantly modulating host cell viability upon *S. aureus* USA300 infection. Candidate selection was refined by network centrality (degree > 50) and literature-based relevance, resulting in 10 prioritized miRNAs for validation.

Supplementary Table S3 Differential gene expression profile following miR-4430 transfection. List of host genes with corresponding base mean expression values, log2 fold changes, standard error (lfcSE), test statistic, raw p-values, and adjusted p-values (padj) obtained from DESeq2 analysis

Supplementary Table S4 Differential gene expression profile following miR-147a transfection. Host genes significantly altered upon transfection with hsa-miR-147a, including base mean, log2 fold change, lfcSE, test statistic, p-value, and adjusted p-value.

Supplementary Table S5 Differential gene expression profile following miR-1249-5p transfection. Summary of host gene expression changes induced by hsa-miR-1249-5p, with DESeq2-calculated base mean, log2 fold change, lfcSE, statistic, p-value, and adjusted p-value.
