# Supplementary material for: Host-directed microRNA-based intervention against intracellular Staphylococcus aureus: high-throughput screening identifies miR-4430, miR-147a, and miR-1249-5p as multifunctional antimicrobial candidates
Source: Front Cell Infect Microbiol. 2026 Mar 25;16:1772100. doi: 10.3389/fcimb.2026.1772100 (PMC13057319; doi:10.3389/fcimb.2026.1772100)
Supplement: Supplementary file 1 [file Image1.pdf]

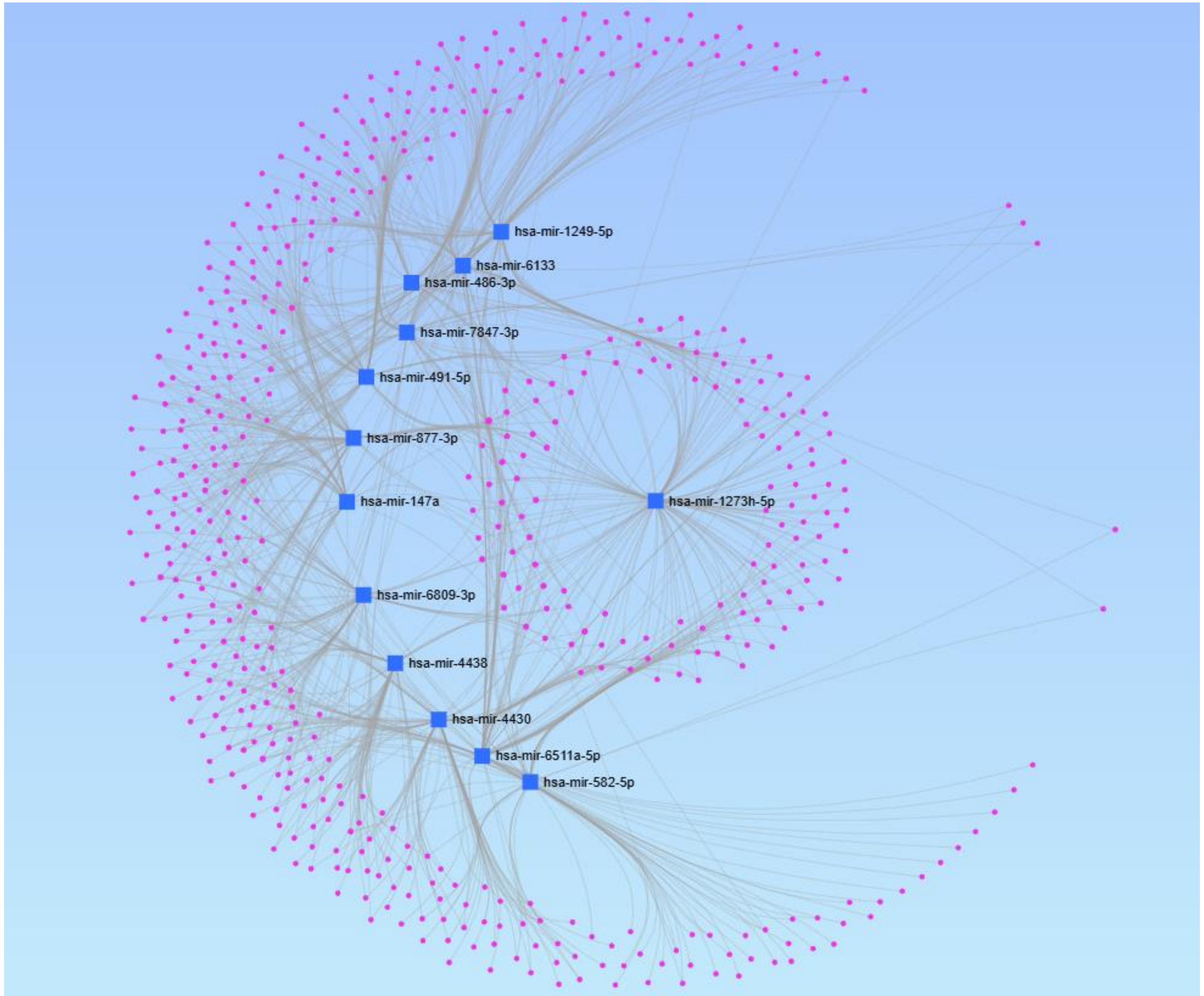

Figure S1. Network centrality analysis of 113 significant miRNAs using the miRNet 2.0 platform. Candidates with degrees < 50 were excluded, resulting in 13 miRNAs (Table S1). Further refinement based on assay robustness and infection-related literature reduced the list to 10 candidates selected for downstream functional validation studies. Together, these results identified ten highly connected miRNAs with potential host-directed antimicrobial activity against intracellular *S. aureus*.

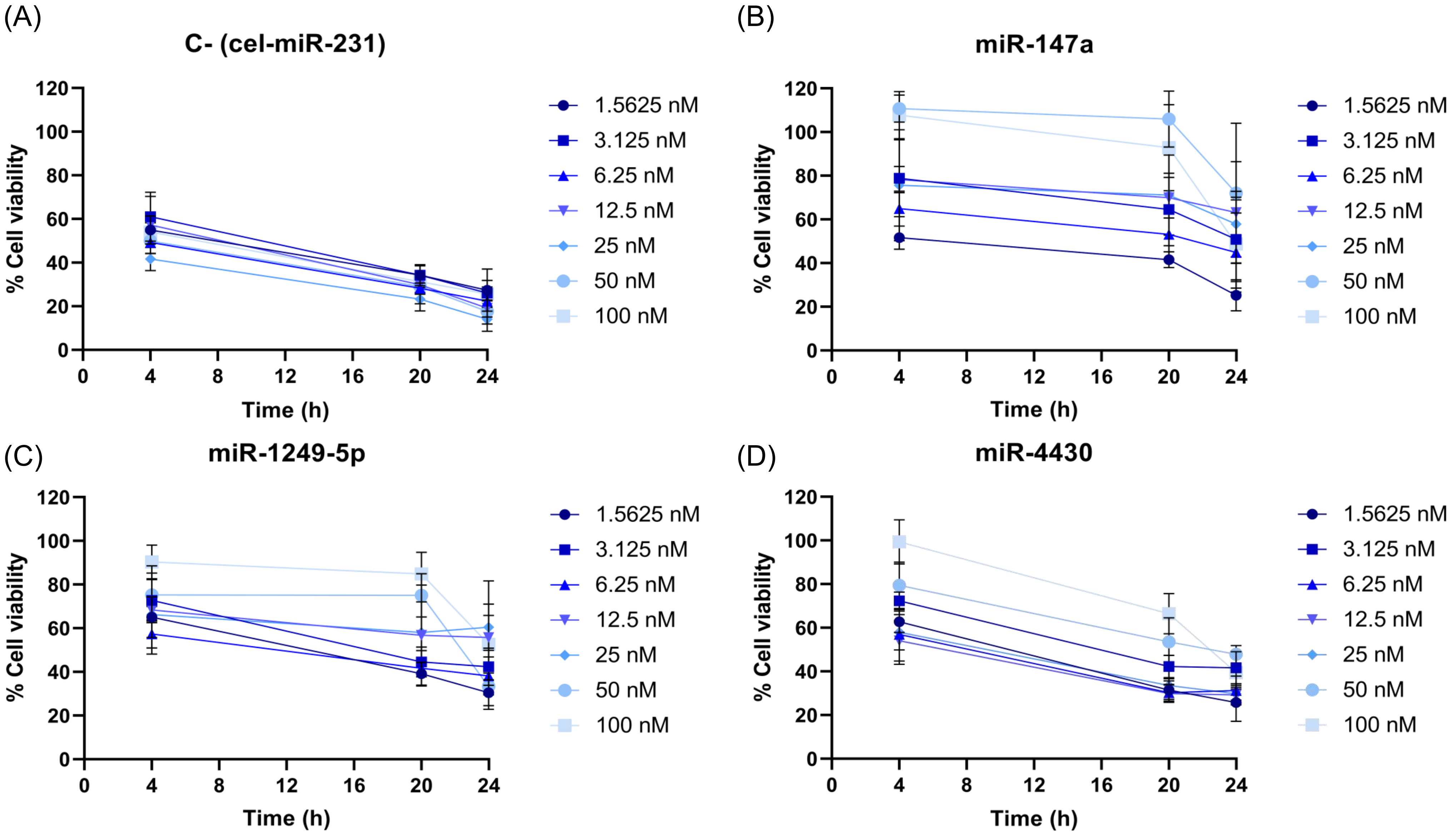

Figure S2. Dose–response analysis reveals a transient host cell viability–preserving effect of selected miRNAs during *S. aureus* infection. A549 epithelial cells were transfected with increasing concentrations of individual miRNA mimics and subsequently infected with *S. aureus* USA300 (MOI = 10). Host cell viability was assessed at early and late time points post-infection, including 20 h post-infection, to evaluate the persistence of the miRNA-mediated effect. Several miRNAs displayed a dose-dependent preservation of host cell viability at earlier time points; however, this effect was not sustained, and a pronounced reduction in viability was observed at 20 h post-infection across all tested conditions. Data represent four independent biological replicates (n = 4).

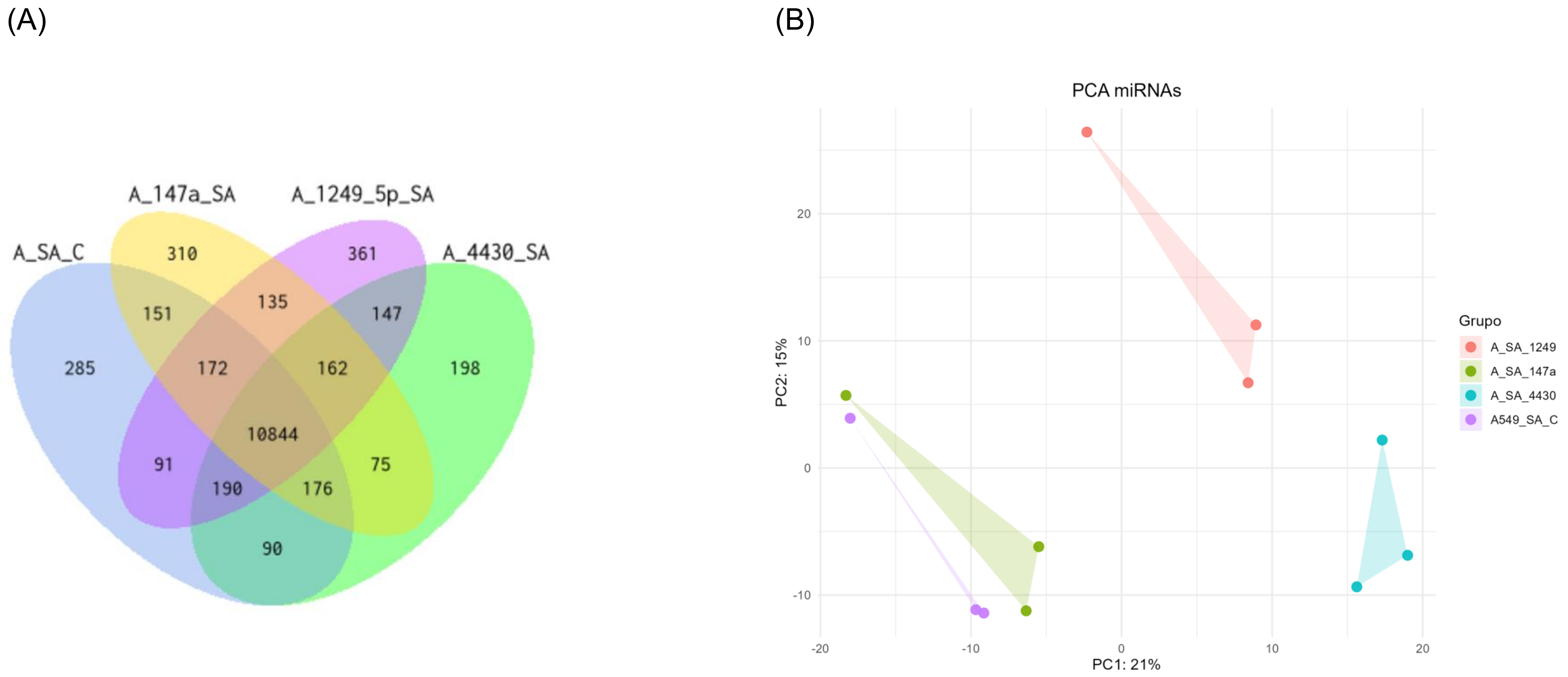

Figure S3. Transcriptomic impact of miRNA treatment on host cells infected with *S. aureus* USA300. (A) Number of uniquely expressed genes in A549 cells transfected with miR-4430, miR-1249-5p, or miR-147a prior to infection. miR-1249-5p induced the highest number of unique transcriptional changes. (B) Clustering of transcriptomic profiles showing tighter co-regulation of gene expression in miR-4430–transfected cells than in miR-1249-5p and miR-147a. These results indicate that each miRNA exerts a distinct transcriptional signature, with miR-1249-5p producing broader effects and miR-4430 exhibiting more consistent gene regulation.

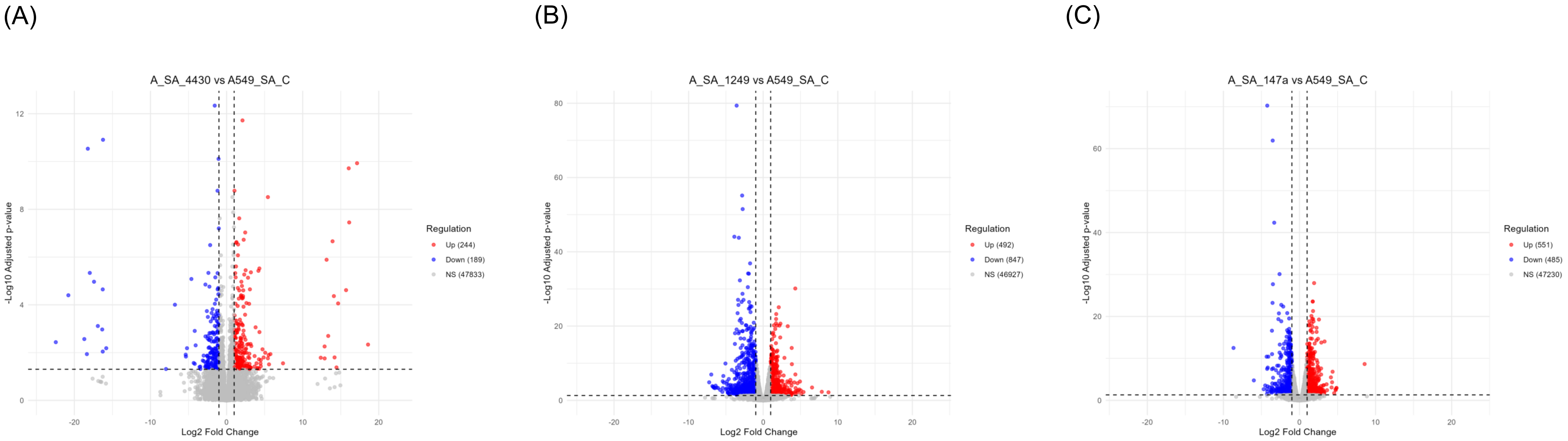

Figure S4. Host differentially expressed genes following miRNA transfection and *S. aureus* USA300 infection. Volcano plots showing differentially expressed host genes in A549 cells transfected with miR-4430 (A), miR-1249-5p (B), and miR-147a (C) and subsequently infected with *S. aureus* USA300. Differential expression was determined relative to infected control cells transfected with control miRNA. Plots display $\log_2$ fold change versus $-\log_{10}$ adjusted p-value. These data provide an overview of miRNA-associated host transcriptional responses under infection conditions.

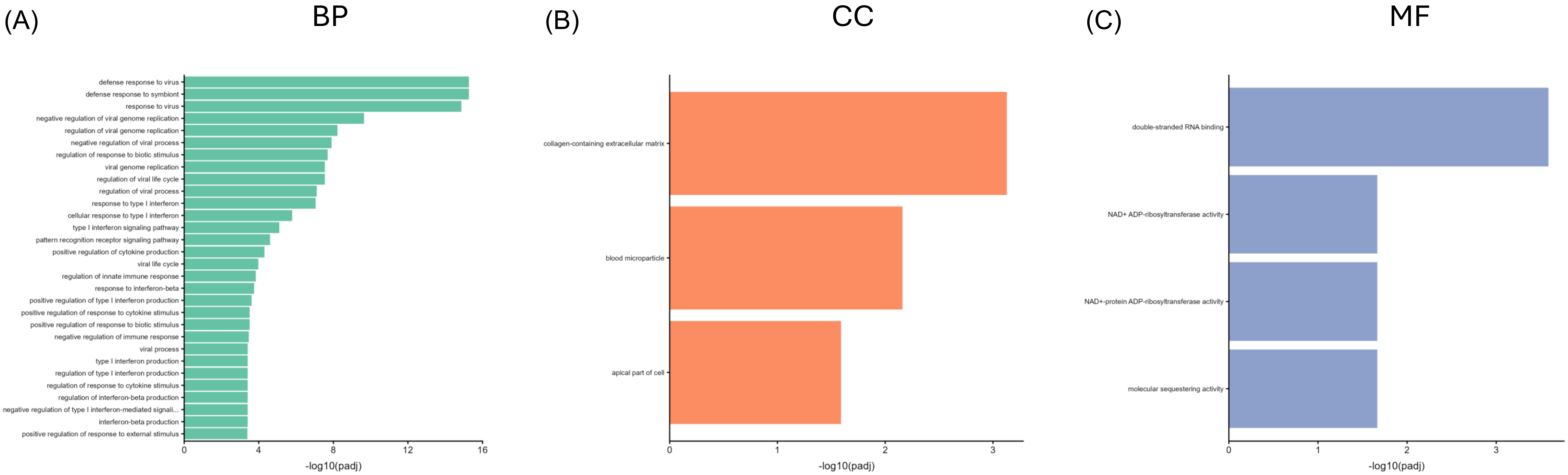

Figure S5. GO enrichment analysis of host transcriptomic changes induced by miR-4430 during *S. aureus* infection. (A) Biological Process (BP) terms enriched in A549 cells transfected with miR-4430, including immune-related pathways such as defense response to virus, type I interferon signaling, and cytokine production. (B) Cellular Component (CC) terms enriched, notably blood microparticles, which are linked to immune modulation. (C) Molecular Function (MF) terms enriched, reflecting the activation of immune regulatory mechanisms. These analyses indicate that miR-4430 enhances host immune responses, particularly interferon-related pathways, thereby contributing to the intracellular control of *S. aureus*.

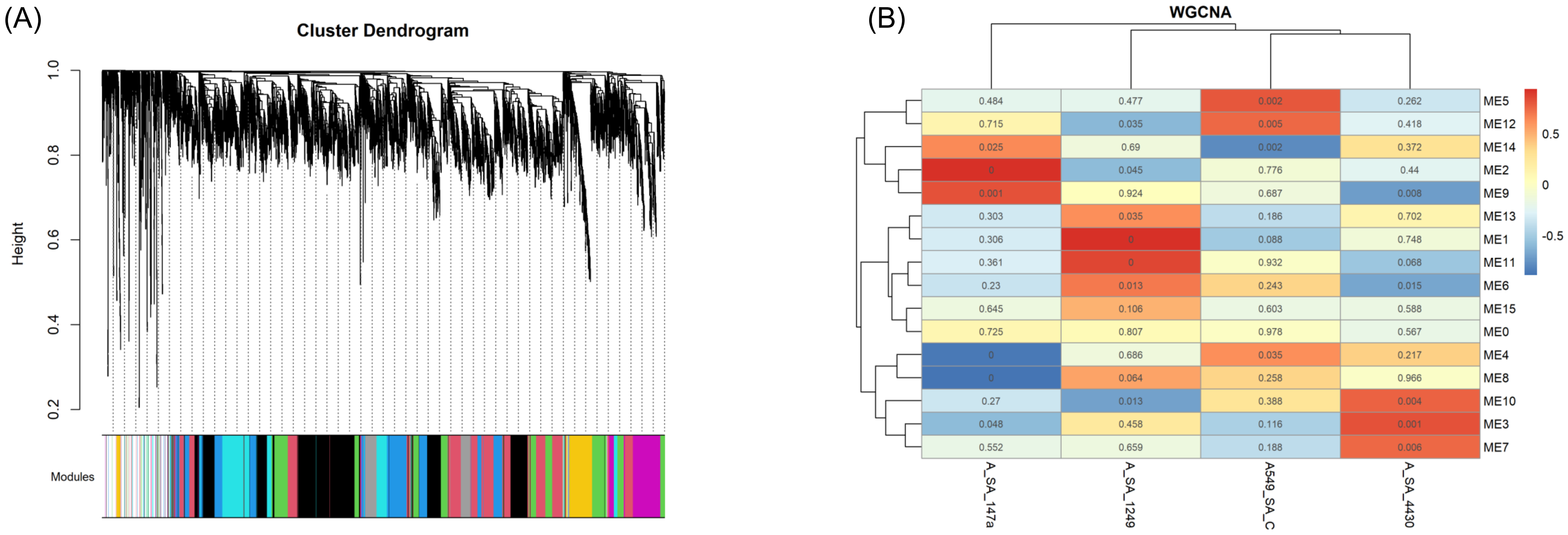

Figure S6. WGCNA of host responses to miR-4430, miR-147a, and miR-1249-5p during *S. aureus* infection. (A) Module–trait correlation heatmap showing the association of specific co-expression modules with each miRNA treatment in infected A549 cells. (B) Representative gene modules enriched under each condition, highlighting immune-related modules for miR-4430, adhesion- and extracellular matrix–related modules for miR-147a, and metabolic/stress-related modules for miR-1249-5p. These results demonstrate that each miRNA is linked to distinct co-expression networks, consistent with their complementary regulatory mechanisms during intracellular *S. aureus* infection.

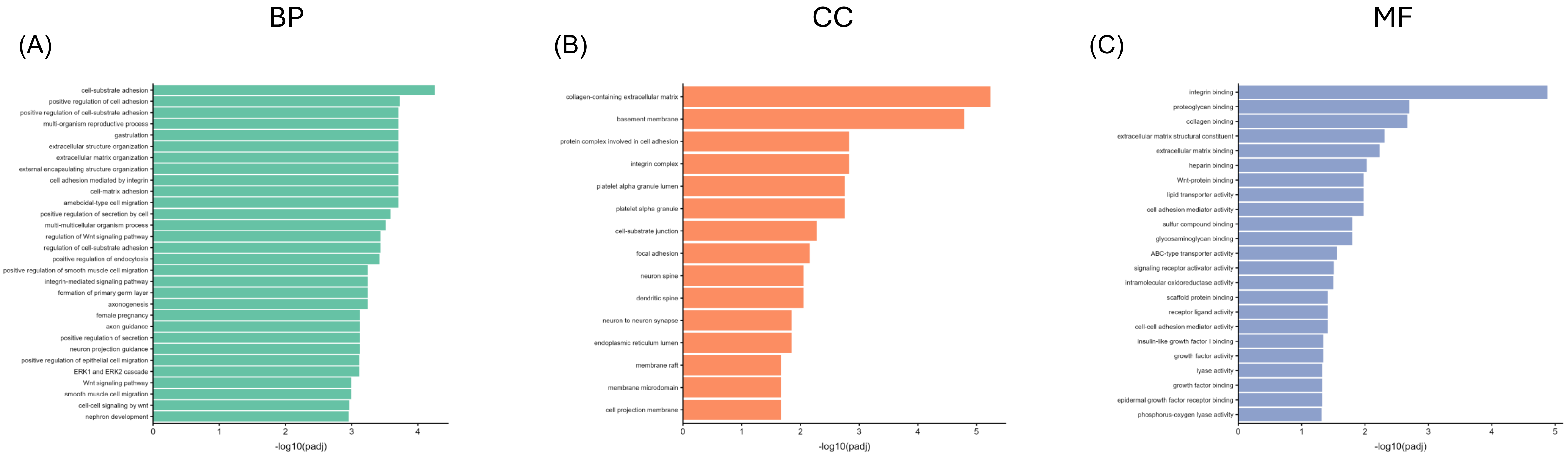

Figure S7. GO enrichment analysis of host transcriptomic changes induced by miR-147a during *S. aureus* infection. (A) Biological Process (BP) terms enriched, including extracellular matrix organization and cell–substrate adhesion. (B) Cellular Component (CC) terms enriched, such as collagen-containing extracellular matrix and adhesion-related protein complexes. (C) Molecular Function (MF) terms enriched, notably integrin and proteoglycan binding. These results show that miR-147a regulates pathways linked to extracellular matrix remodeling and integrin-mediated adhesion, mechanisms that are directly involved in *S. aureus* internalization.

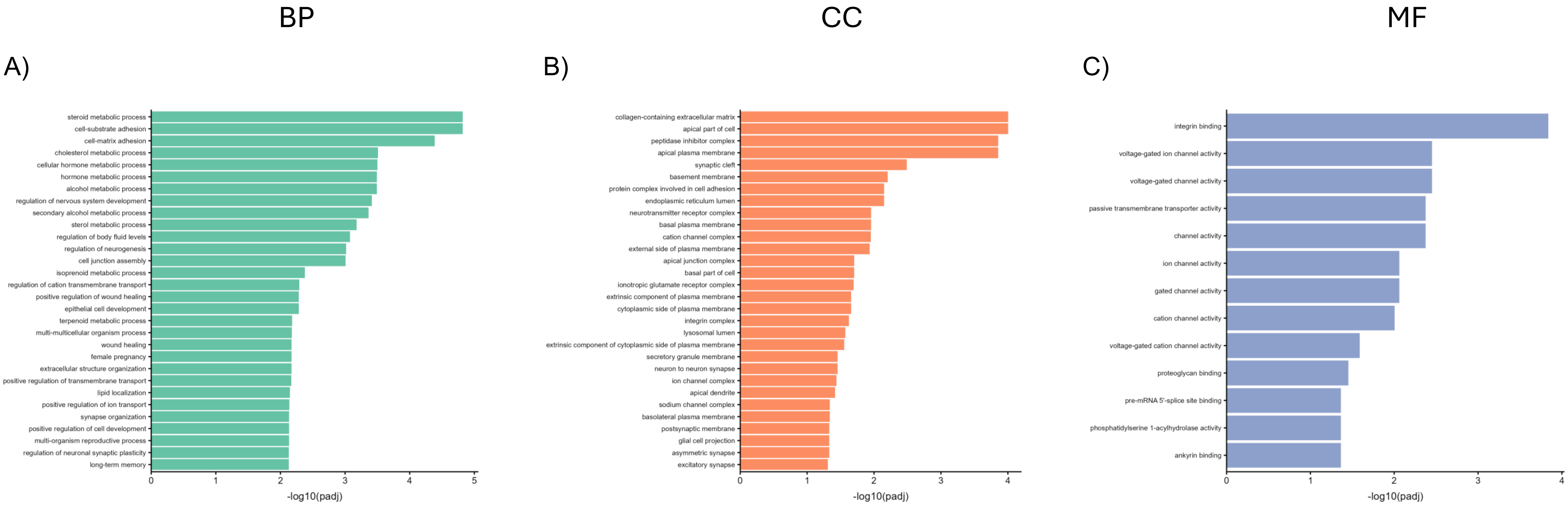

Figure S8. GO enrichment analysis of host transcriptomic changes induced by miR-1249-5p during *S. aureus* infection. (A) Biological Process (BP) terms enriched, including cell–substrate adhesion and steroid metabolism. (B) Cellular Component (CC) terms enriched, such as extracellular matrix organization and cell surface structures. (C) Molecular Function (MF) terms enriched, reflecting integrin binding and adhesion-related interactions. These findings suggest that miR-1249-5p modulates extracellular matrix organization and lipid-associated processes, consistent with the regulation of membrane domains important for *S. aureus* uptake.

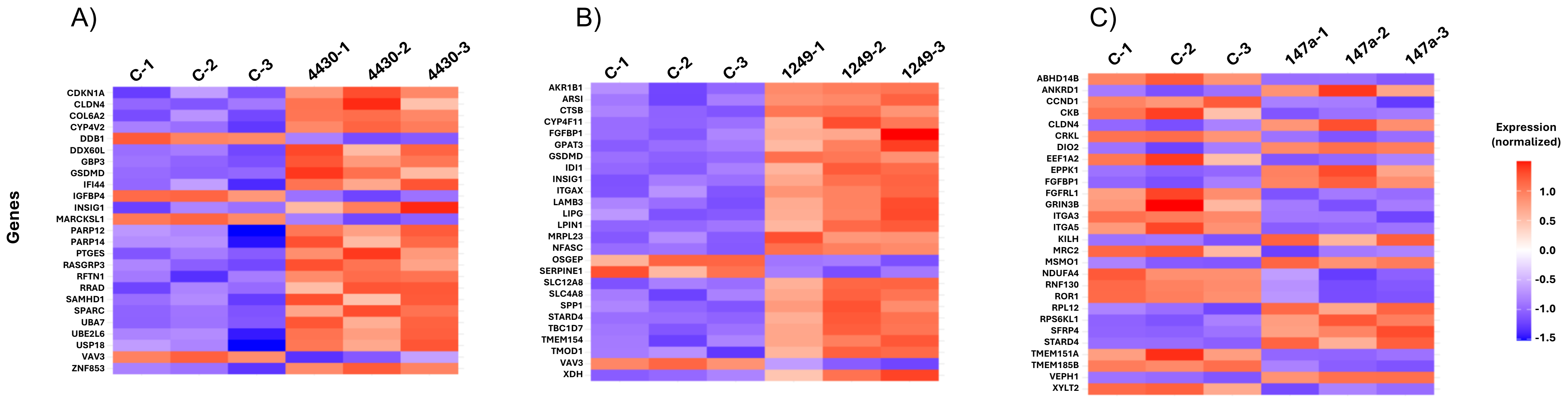

Figure S9. Heatmaps showing the most differentially expressed host genes in A549 cells infected with *S. aureus* USA300 and transfected with miR-4430 (A), miR-1249-5p (B), or miR-147a (C). Heatmaps represent scaled expression values (row-wise Z-scores) of the top differentially expressed genes identified by RNA-seq for each condition relative to infected, non-transfected controls. Each column corresponds to an independent biological replicate, and each row represents an individual gene. Color gradients indicate relative expression levels, with red denoting higher expression and blue denoting lower expression.

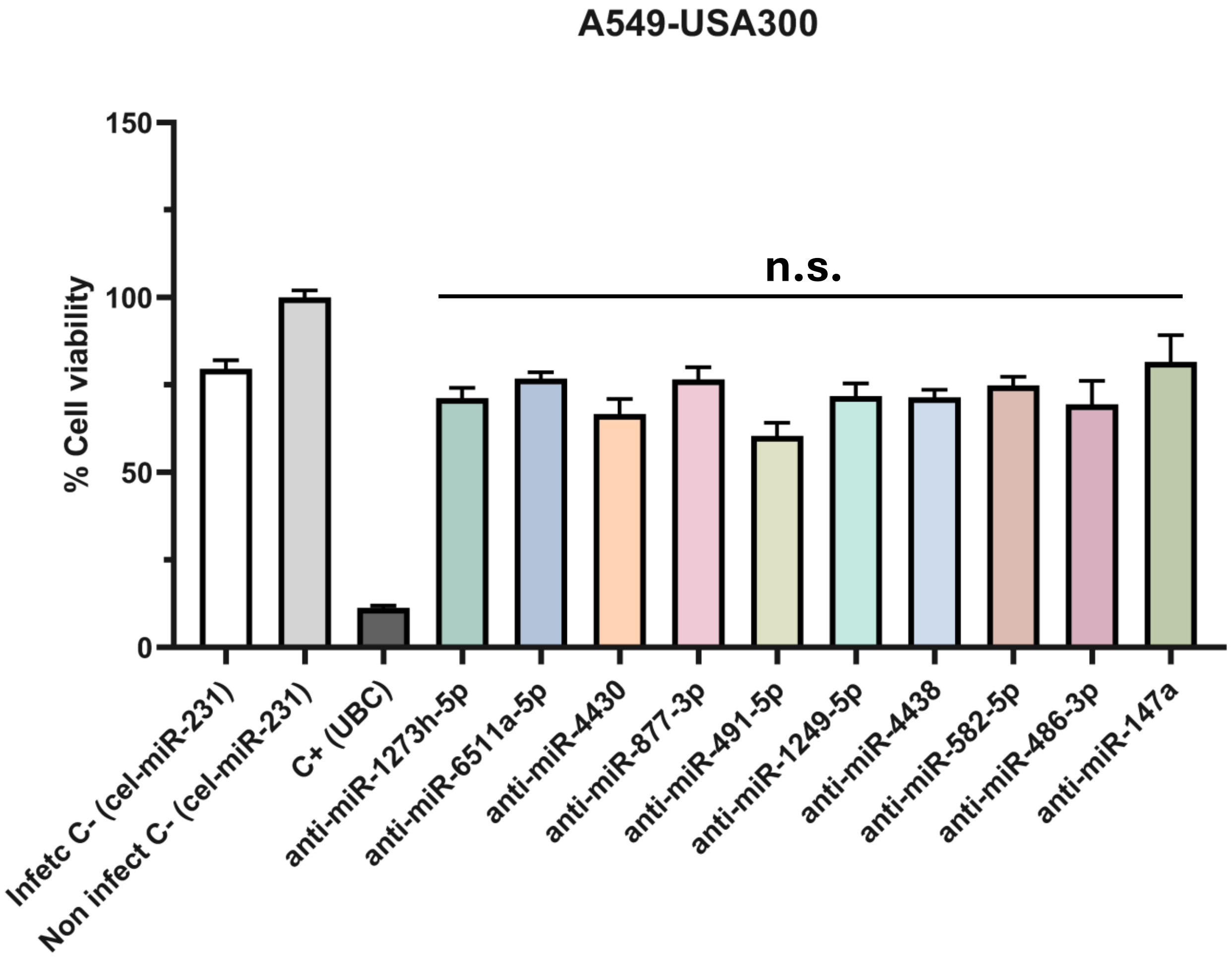

Figure S10. Antagomir-mediated inhibition of selected miRNAs does not significantly alter host cell viability during *S. aureus* infection in A549 cells. A549 epithelial cells were transfected with individual antagomirs targeting selected human miRNAs and subsequently infected with *S. aureus* USA300 at a multiplicity of infection (MOI) of 5. Host cell viability was assessed 20 h post-infection and expressed as percentage relative to non-infected controls. Data represent four independent biological replicates (n = 4). No statistically significant differences in cell viability were observed between cells transfected with any antagomir and the infected cells transfected with a non-targeting *C. elegans* miRNA control (cel-miR-231), indicating that inhibition of these individual miRNAs does not exacerbate infection-induced cytotoxicity under the conditions tested. N.s., non-significant.

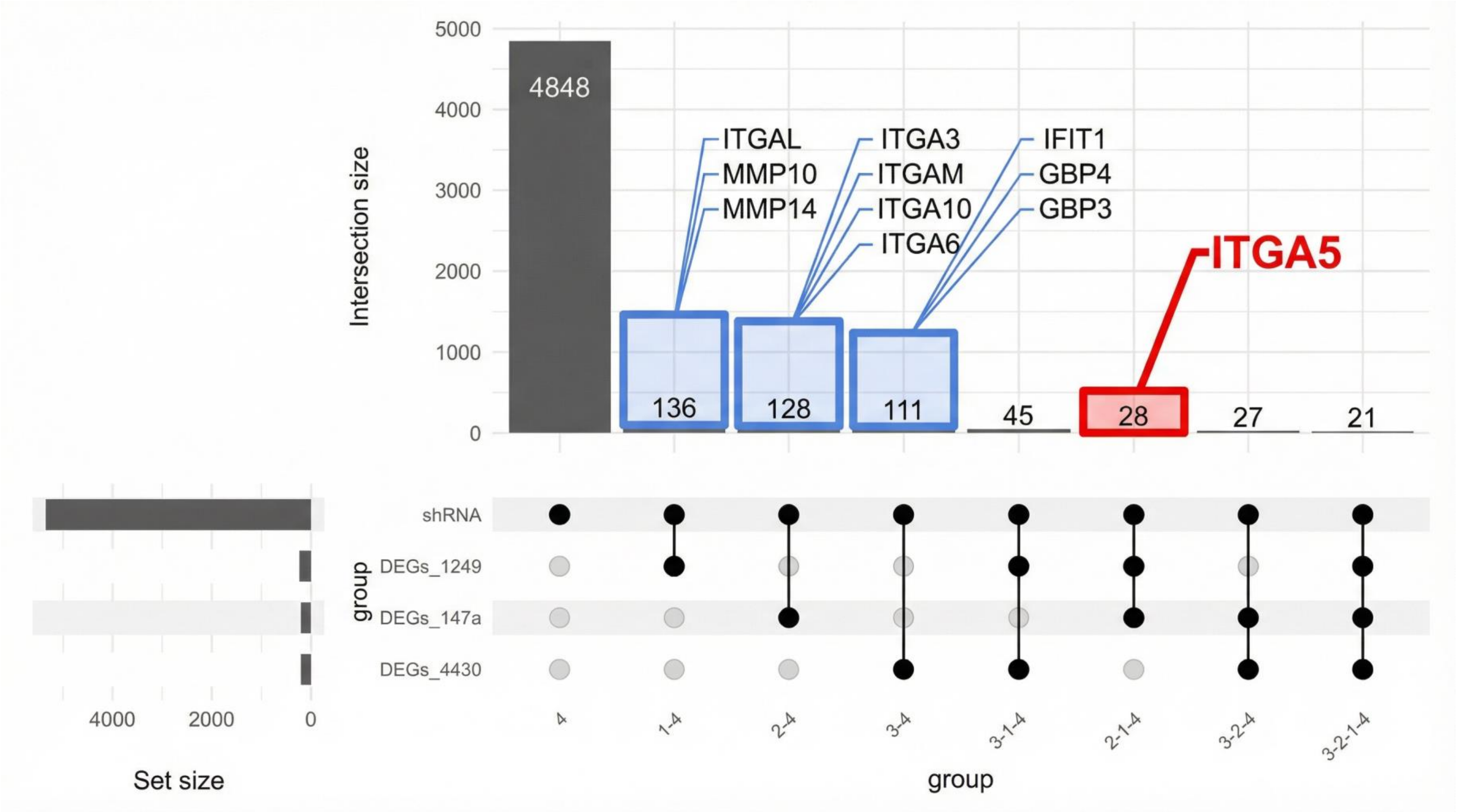

Figure S11. Integration of shRNA screening and miRNA RNA-seq datasets using an UpSet plot. UpSet plot depicting the overlap between differentially expressed genes (DEGs) identified in A549 cells transfected with miR-4430, miR-147a, or miR-1249-5p during *S. aureus* infection and a high-confidence set of genes supported by shRNA screening. shRNA targets were filtered based on reproducibility across replicates, statistical significance, and effect size to retain robust candidates. Bars indicate the size of each intersection, while the matrix below denotes the specific combinations of datasets contributing to each intersection. The complete filtered shRNA gene set is included as a reference to contextualize the relative size of condition-specific overlaps.
