# Supplementary material for: Host-directed microRNA-based intervention against intracellular Staphylococcus aureus: high-throughput screening identifies miR-4430, miR-147a, and miR-1249-5p as multifunctional antimicrobial candidates
Source: Front Cell Infect Microbiol. 2026 Mar 25;16:1772100. doi: 10.3389/fcimb.2026.1772100 (PMC13057319; doi:10.3389/fcimb.2026.1772100)
Supplement: Supplementary Table S2 — List of miRNAs identified in the high-throughput screen as significantly modulating host cell viability upon S. aureus USA300 infection. Candidate selection was refined by network centrality (degree > 50) and literature-based relevance, resulting in 10 prioritized miRNAs for validation. [file Table2.docx]

| miRNA | Degree | Betweenness |
| --- | --- | --- |
| hsa-mir-1273h-5p | 131 | 29575.04 |
| hsa-mir-6511a-5p | 102 | 18918.34 |
| hsa-mir-4430 | 84 | 16144.91 |
| hsa-mir-877-3p | 80 | 16094.82 |
| hsa-mir-491-5p | 73 | 11201.99 |
| hsa-mir-6133 | 73 | 10049.31 |
| hsa-mir-1249-5p | 68 | 9024766 |
| hsa-mir-4438 | 63 | 10341.96 |
| hsa-mir-6809-3p | 59 | 9593578 |
| hsa-mir-7847-3p | 59 | 7670764 |
| hsa-mir-582-5p | 54 | 7634494 |
| hsa-mir-486-3p | 54 | 7114791 |
| hsa-mir-147a | 50 | 5777016 |
